# Is Smokeless Tobacco Use an Appropriate Public Health Strategy for Reducing Societal Harm from Cigarette Smoking?

**DOI:** 10.3390/ijerph6010010

**Published:** 2008-12-23

**Authors:** Scott L. Tomar, Brion J. Fox, Herbert H. Severson

**Affiliations:** 1 University of Florida College of Dentistry, Gainesville, 1329 SW 16th Street, Suite 5180 P.O. Box 103628, Gainesville, FL 32610-3628 USA FL, USA; 2 University of Wisconsin Comprehensive Cancer Center, #385 Warf Office Building, 610 Walnut Street, Madison, WI 53726 USA; E-Mail: bjfox@uwccc.wisc.edu; 3 Oregon Research Institute, 1715 Franklin Blvd., Eugene, OR 97403 USA; E-Mail: herb@ori.org

**Keywords:** Smokeless tobacco, harm reduction, smoking cessation, smoking initiation

## Abstract

Four arguments have been used to support smokeless tobacco (ST) for harm reduction: (1) Switching from cigarettes to ST would reduce health risks; (2) ST is effective for smoking cessation; (3) ST is an effective nicotine maintenance product; and (4) ST is not a “gateway” for cigarette smoking. There is little evidence to support the first three arguments and most evidence suggests that ST is a gateway for cigarette smoking. There are ethical challenges to promoting ST use. Based on the precautionary principle, the burden of proof is on proponents to provide evidence to support their position; such evidence is lacking.

## 1. Introduction

There is increasing interest and controversy within the public health community about smokeless tobacco (ST) [1]. Some proponents believe that, given the enormity of the health problems associated with cigarette smoking, a viable alternative is harm reduction, i.e., use of nicotine-containing products with lower mortality and morbidity risks [2, 3]. This has resulted in recommendations for cigarette smokers to switch to ST products [4–7].

A recent review article claimed “there is a strong scientific and medical foundation for tobacco harm reduction, and it [ST use] shows great potential as a public health strategy to help millions of smokers [4, p. 17]. One group of advocates for ST-based harm reduction characterized their opponents’ position as “health professional’s authoritarian insistence that the only valid choice for smokers is to quit or die as an addicted cigarette user.” [8, p. 363].

What are the arguments put forward by proponents of ST use and is there sufficient evidence to support them? We had three purposes for this paper: (1) to identify the major arguments used by those who support ST as a tobacco harm reduction strategy; (2) to summarize and critique the scientific evidence behind those arguments; and (3) to consider the ethical and practical implications of promoting ST use for population harm reduction. In this paper, we will be focusing mainly on research from the United States and international findings that may or may not be relevant for ST use in the United States, keeping in mind that the context and products from other countries may not necessarily be transferable to the current situation in the United States. We will be addressing arguments that have been made about the use of ST, as well as describing some recent events and behaviors by U.S. tobacco companies.

## 2. Background

The common feature of smokeless tobacco products is that they are not burned when used. In North America and parts of Europe, ST most commonly comes in the form of snuff (dry or moist) or chewing tobacco. Moist snuff, used orally, is the most popular form of ST [9]. Although having a much smaller market than cigarettes, ST products account for about $2.6 billion in sales annually in the United States [10]. ST use in the United States occurs predominantly among males, with higher prevalence among younger-aged whites, rural residents, and in some Native American and Alaska Native tribes [9]. Similarly, ST use in Sweden and Norway occurs largely among males [11, 12]. In 2005, an estimated 6% of men and less than 1% of women in the United States used ST [13].

There is scientific consensus that ST use increases the risk for cancers of the oral cavity and pharynx, oral soft tissue lesions, gingival recession, and nicotine addiction [14–18]. Recent Swedish, Norwegian, and U.S. studies found an increased risk for pancreatic cancer from using ST [19–21]. ST has been classified as a human carcinogen by the International Agency for Research on Cancer [22, 23], the U.S. Surgeon General [24], and the National Toxicology Program [25]. Some ST proponents suggest that the health risks associated with ST use are greatly exaggerated [4], particularly for moist snuff products with lower levels of tobacco-specific nitrosamines (TSNAs) such as Swedish snus [26]. The United States currently has no regulatory framework or standards for levels of TSNAs or other toxins in ST products, and their levels are substantially greater in most U.S. moist snuff products than in products sold in Sweden [27, 28].

## 3. Current situation

Two recent circumstances have increased attention on ST. First, there is growing consideration of ST use within the public health community. Several recent publications indicate that ST is being taken more seriously by scientists as a harm reduction strategy [3, 17, 26, 29].

Second, the two largest U.S. cigarette manufacturers have entered the ST market. In 2006, Reynolds American purchased Conwood, a major ST manufacturer with 32% of the U.S. moist snuff market, and began test-marketing Camel Snus [30]. Also in 2006, Philip Morris USA began test-marketing Taboka, an ST product in a pouch [31], and began test marketing Marlboro Snus [32]. In 2008, Philip Morris USA acquired U.S. Smokeless Tobacco Company, the country’s major manufacturer of moist snuff (64% of the US moist snuff market), and expanded test marketing of new moist snuff products. Promotions for these newer ST products focus on their being “spit free” and their use in indoor areas where smoking is not allowed [33, 34].

Some tobacco companies disseminate statements by those who favor ST for tobacco harm reduction, and criticize opponents as being unprincipled in their resistance to harm reduction [35].

## 4. Arguments used to support ST as a harm reduction strategy

Proponents present four major arguments for ST as a harm reduction strategy, each of which is reviewed below.

### 4.1. Smokers Reduce Their Health Risks by Switching to Smokeless Tobacco

The first argument is that, although ST has health risks, the risks are considerably lower than those associated with cigarette smoking. Two broad lines of research are needed to support this argument: (a) exclusive ST users who never smoked cigarettes experience fewer health risks than exclusive smokers; and b) exclusive smokers who switch to only using ST experience reduced health risks compared to those who continue to smoke.

There is substantial evidence that lifetime ST-only users have lower health risks than lifetime cigarette-only smokers [15, 16, 36, 37]. For example, unlike smoking, ST products do not appear to increase the risk for lung cancer or chronic obstructive pulmonary disease. Some have attempted to quantify the reduced level of health risks among ST-only users compared with cigarette-only users [4, 38].

Proponents also stress that not all ST products are the same. They argue that moist snuff, particularly lower-TSNA products, has fewer health risks than dry snuff or chewing tobacco [4]. Population-based data from men in Sweden, a country with a high prevalence of ST and a low prevalence of daily cigarette smoking, are cited to demonstrate lower rates of smoking-related cancers compared to men in other European countries with different patterns of tobacco use [39, 40].

We found little evidence that lifetime cigarette-only users who switch to ST reduce their health risks compared to continuing smokers, although it is very unlikely that smokers who completely switched to using ST would increase their health risks. Only one published study compared mortality rates among smokers who permanently switched to ST with those who quit smoking entirely. An analysis of data from American Cancer Society’s second cancer prevention study (CPS II) cohort [37] found that men who switched from cigarette use to using only ST had higher death rates from all causes combined, lung cancer, coronary heart disease, or stroke compared with those who had quit smoking completely. Mortality rates from all causes among “switchers” appear to be lower than for continuing smokers in CPS-II [41].

An extension of the “ST is safer” argument is that individuals should be provided with information on the comparative health risks of products, weigh the risks, and decide for themselves [42]. Proponents recommend providing “objective scientific data,” and criticize health organizations and governmental agencies for exaggerating ST health risks and implying its use is “just as dangerous as smoking” [4, 43]. Unfortunately, smokers may substantially underestimate their current risks [44] and decades of research indicates that scientific number-based messages alone are not effective in changing smoking or other behaviors [45, 46].

Although health risks from using only ST are lower than those for exclusively smoking, this comparison of health risks is true for virtually any consumer product. Proponents do not compare ST risks with risks associated with FDA-approved smoking cessation pharmaceuticals or with the lower risks associated with quitting tobacco entirely [37]. If these types of risk comparisons were made, the health risks for ST would not seem small. In addition, proponents do not address the health effects of multiple tobacco product use, although the prevalence of ST used in combination with other tobacco products may be substantial. U.S. national data indicate that 40% of men who occasionally used snuff and 19% who used snuff daily also smoked cigarettes [47], and 26% of male cigarette smokers in 10 states also used other forms of tobacco [48].

In summary, we conclude that exclusive use of ST confers fewer health risks than exclusive cigarette smoking. However, ST use has important health risks that are substantially greater than those associated with not smoking. We identified one study demonstrating that smokers who switched to using only ST reduced their risks for fatal health outcomes, although that study also found slightly higher mortality rates among lifetime cigarette-only smokers who switched to ST compared with those who quit tobacco use entirely.

### 4.2. ST Use is Effective for Smoking Cessation

The second claim is that the ST is an effective smoking cessation aid. Various approaches have been used to estimate the extent to which ST has been used as a smoking cessation method from survey data: specific questions about cessation methods used; the proportion of current ST-only users who were former cigarette smokers; reconstructed birth cohorts using serial surveys; and comparative population trends in prevalence for cigarette smoking and ST use.

ST proponents cite the experience in Sweden to support use of ST (snus) for smoking cessation. Although overall tobacco use has changed little over the past 20 years in Sweden, smoking prevalence among men and women declined while ST use by men increased, with the implication being that some of the reduction in smoking prevalence resulted from smokers switching to using ST [4, 49–51]. However, that pattern has not been found in other countries. In Norway, for example, the prevalence of ST use among males aged 16–24 years increased from 9% in 1985 to 21% in 2002, while the prevalence of cigarette smoking remained relatively constant [52].

Survey data on the use of ST as a method for quitting smoking are available from the U.S. and Sweden. An estimated 7% of men in a 1986 U.S. national survey who were former cigarette smokers used ST for quitting [53]. In a 2001–2002 national Swedish survey, ST was reportedly used in the last quit attempt by 24% of men who smoked cigarettes, making it the most common cessation aid used by that population [51].

Analyses of U.S. data from the 1998 National Health Interview Survey (NHIS) found that about 6% of daily snuff users reported having quit cigarette smoking within the preceding year, suggesting that they had switched to ST [52]. Two groups of researchers used data from the 1987 and 2000 NHIS and offered contrasting interpretations [54, 55]. Kozlowski *et al.* [54] found that among men aged 22–34 years, those who smoked cigarettes but became snuff users were twice as likely as never users of snuff to have quit smoking. On the other hand, Tomar and Loree [55] analyzed male birth cohorts and found that about 1% of former cigarette smokers aged 36–47 years had used snuff or chewing tobacco to quit smoking, although about 19% had been regular ST users at some time.

Intervention trials provide more direct evidence of the effectiveness of ST use for smoking cessation. To our knowledge, only two trials have been conducted in which ST was used for cessation. An uncontrolled U.S. trial reported results for one- and seven-year follow-ups [56, 57]. Sixteen of 63 subjects (25%) had used snuff and successfully quit smoking at the 1-year follow-up; at the 7-year follow-up, 12 of 16 persons who had used ST remained abstinent from smoking (an overall quit rate of about 19%). A recently reported open label randomized trial conducted in Denmark included smokeless tobacco and group support in the intervention arm and only group support in the control arm [58]. That study found significantly greater point prevalence and continuous abstinence rates in the ST group than in the control group at 7 weeks, but no significant difference between groups in 6-month point prevalence abstinence rates (23.1% vs. 20.8%). The authors concluded that the trial demonstrated short-term efficacy of ST in combination with group support for smoking cessation but no long-term efficacy.

In summary, while data from Sweden suggest that ST is a cessation aid for some cigarette smokers, findings from U.S. studies are inconsistent; it is clear, however, that ST is not widely used for smoking cessation in the United States. The only known randomized trial of ST use for smoking cessation found no long-term efficacy. We conclude that, at present, there is insufficient evidence for ST as an effective cigarette smoking cessation aid.

### 4.3. ST is an Effective Nicotine Maintenance Product for Highly Nicotine-dependent (“Hard Core”) Smokers

The third argument is based on two assumptions: ST use has lower risks than cigarette smoking, and some people who are unwilling or unable to quit using tobacco by evidence-based approaches [59] could use ST for long-term nicotine maintenance. The rationale is similar to that used for reducing harm from the abuse of other substances: long-term substitution of a less dangerous product is more desirable than continued use of a more dangerous product. A model similar to this is the long-term treatment of heroin addiction with methadone [60]. ST proponents note that FDA-approved nicotine-containing products for smoking cessation are not approved for long-term nicotine maintenance, and that the bioavailability of nicotine from ST is greater than that achieved for FDA-approved nicotine products [4]. They point out that opponents of substituting ST for cigarettes offer no help for “hard core” smokers who want to reduce their health risks, i.e., the only choices are to “quit or die” [8]. Proponents of nicotine maintenance believe that ST would provide a “middle way” to help smokers reduce their health risks [4]. The argument continues that, although at some point persons using ST for nicotine maintenance ideally would decide to quit ST use entirely, even if they never quit ST use their health risks would be lower than if they continued to smoke cigarettes.

There are three problems with this argument. First, unlike methadone, we found no studies that examined the rates of relapse to cigarette smoking for person using ST for long-term nicotine maintenance, either alone or in comparison to FDA-approved pharmacotherapies. Thus, the relative effectiveness of ST as a long-term nicotine maintenance strategy is unknown. Second, harm reduction treatments for heroin use are managed and monitored under the guidance of health care professionals, as these treatments require prescriptions. In contrast, ST products are available “over-the-counter,” are still heavily marketed to young males and have a high potential for abuse, and have levels of bioavailable nicotine that vary widely by product [61, 62]. Third, the mechanism by which methadone works is completely different from ST: methadone operates to reduce or eliminate withdrawal symptoms by blocking receptors [60] In contrast, ST contains the same addictive substance (nicotine) as cigarettes, which may continue nicotine addiction and perpetuate relapse.

There is strong support for the statement that some people are more addicted than others to nicotine, which makes smoking cessation more difficult for them [15]. Some ST products have higher and more rapid nicotine dosing than some FDA-approved nicotine replacement products [4]. Although ST proponents may intend to help smokers who “have tried everything” but have been unable to quit, encouraging ST use for long-term nicotine maintenance may delay or prevent persons who would otherwise end their nicotine addiction from doing so [63]. We conclude there is currently no evidence on the effectiveness of ST for long-term nicotine maintenance.

### 4.4. ST use is not a “Gateway” Product for Initiating Cigarette Smoking

The final argument is that ST users are not at increased risk of initiating cigarette smoking, or that ST use may even prevent smoking initiation [8, 49, 51]. The not-a-gateway argument is crucial for ST proponents in order to support the first three arguments, because if ST use increased rates of smoking initiation there is no rationale for advocating its use as a harm reduction product.

Research to support the not-a-gateway argument comes from several types of studies. Indirect evidence, based on comparative prevalence trend data for cigarette smoking and ST use among adolescents and adults in Sweden over the past 20 years, are cited to support this position: if ST is a gateway to cigarette smoking, then smoking prevalence would not decrease at the same time ST use increased [4].

Comparative population-based prevalence trend data for ST use and cigarette smoking are not supported by data from countries other than Sweden. In the United Stated, trends in ST use and cigarette smoking among adolescent boys and men tended to parallel each other during the past two decades, with a drop in use of both types of tobacco products among boys since 1997 [64]. Norway, where ST use by young males has increased sharply during the past two decades, did not experience a decline in smoking initiation during most of that period [52].

Using data from the 1987 NHIS, Kozlowski *et al.* [54] examined age of first use by type and sequence of use of different tobacco products. More than three-quarters of males aged 23–34 were classified as non-gateway users; i.e., use of snuff did not precede use of cigarettes. However, several U.S. prospective cohort and cross-sectional studies of young males found that use of ST was predictive of cigarette smoking initiation [65–68].

More recent U.S. studies provide additional support for ST use as a predictor of cigarette smoking initiation. Using data from the 1989 and 1993 national Teenage Attitudes and Practices Survey (TAPS), Tomar [69] found that adolescent males who were ST-only users were more than three times as likely as never ST-users to become cigarette smokers within the following four years. A re-analysis of the TAPS data by O’Connor et al. [70] concluded that regular ST use was not a statistically significant predictor of current smoking when psychosocial risk factors were included in the models. However, those authors equated lack of statistical significance due to the small number of ST users at baseline and highly parameterized regression models with lack of effect [71]. In the TAPS data, the strength of association between ST use and subsequent smoking was on the same order of magnitude as many established psychosocial risk factors for smoking.

Severson *et al.* [72] followed a cohort of adolescent boys in grades seven and nine who were ST-only users for two years. Among the ST-only users, 26% maintained their ST-only status, 17% switched and became cigarette-only users, and 41% became dual users of ST and cigarettes at follow-up. Among nonsmoking, non ST users, 24.0% maintained their status, 15.7% reported using cigarettes and only 8.3% reported using both products. They found that initiation of weekly smoking in grades nine and eleven was significantly associated with baseline ST use, even after controlling for other risk factors.

Haddock *et al.* [73] followed a cohort of almost 8,000 young adult male Air Force recruits who had not smoked in the past year. Both current and former ST users were more than twice as likely as never-users to begin smoking.

In summary, the preponderance of evidence suggests that ST use is a predictor of cigarette smoking in the United States. The findings in one country regarding temporal changes in patterns of tobacco use cannot be assumed to apply elsewhere.

## 5. Discussion

The primary evidence supporting ST use as a harm reduction strategy is that exclusive using ST has lower health risks than does cigarette smoking. There is some evidence that cigarette smokers who switch to ST use may reduce their health risks. There is little evidence that ST use is effective for smoking cessation; or that ST is an effective nicotine maintenance product. In addition, the available evidence suggests that ST use may be a gateway to smoking initiation in the United States.

Proponents of ST-as-harm-reduction argue for differential taxation and emphasis on differences in risk among tobacco products, on the grounds that the public should be moved from cigarettes to less harmful forms of tobacco [4]. Most major tobacco control organizations see all tobacco use as a harmful behavior that provides no net societal benefits, and prefer taxation policies and health messages that discourage the initiation or continued use of any form of tobacco.

There are certainly some individuals who have successfully used ST as an aid to smoking cessation or for long-term nicotine maintenance. From a population-based public health perspective, however, there is a real danger of potential unintended adverse consequences of promoting ST for harm reduction. Of greatest concern is that broader promotion of ST would result in an increase in ST initiation and simply add to or increase cigarette smoking among adolescents and young adults, as apparently was the case in Norway.

A second unintended consequence is that promoting ST for harm reduction may imply that tobacco prevention and control efforts have failed [74]. Although great strides have been made in reducing youth tobacco use, only a small proportion of the available resources are currently being used for tobacco control in most states [75]. Substantial reductions in tobacco use could be achieved if tobacco control programs were fully funded. The promotion of ST for harm reduction by the public health community would divert the existing limited tobacco control resources, which may be better spent on practices demonstrated as effective in reducing smoking [76].

Several ethical issues also must be considered. If products with fewer health risks are available and can perform a function similar to the original product, there is an ethical and, often, statutory or other legal requirement to use the substitute product [77, 78]. Product substitution is a long-recognized strategy in fields such as consumer protection and occupational and environmental health; e.g., using synthetic plastics or cellulose in place of asbestos in building materials or automobile brakes. Although ST use has fewer health risks than does cigarette smoking, several FDA-approved and scientifically established pharmacotherapies are available for smoking cessation [59], which constitute safer substitutes for smoking. In addition, to our knowledge, the public health community has never advocated substitution of an unregulated toxin and human carcinogen when known safer alternatives existed.

A second ethical dilemma could result if changes occurred in risk perception about using ST. Research has shown that “low or minimal risk” health messages, in the context of voluntary decision making or personal health behaviors, are commonly interpreted by the public as meaning “no risk” [79]. The history of the tobacco industry’s development and marketing of filtered, “low-tar,” and “light” cigarettes, which resulted in smokers’ perceptions that these products were safer and reduced their desire to quit [80], should be considered a warning about potential unintended consequences of promoting ST for harm reduction.

A third concern is that mixed messages from the public health and medical communities can be problematic. Lay audiences rely heavily on the expert heuristic when assessing information received from health or other experts [81], preferring consistent interpretations and recommendations about what scientific findings mean and what actions are recommended [82]. A message that encouraged people to “not initiate or continue ST use because of its adverse health effects” juxtaposed with “it’s okay to use ST if you are a smoker and have been unable to quit” could result in confusion among the public.

Probably the biggest ethical challenge concerns the potential role of tobacco companies. Cigarette and ST manufacturers are in the business of helping people to develop and maintain nicotine addiction [83], and promotion of dual product use is one strategy to maintain addiction in the face of increasing smoke-free indoor air laws. This scenario is even more likely now that nearly the entire U.S. smokeless tobacco market is controlled by cigarette manufacturers. Many ST users also use other tobacco products, particularly cigarettes [44], and ST products are advertised to smokers for situational use when they cannot smoke due to smoke-free policies [31, 32, 84]. Although many proponents of ST for tobacco harm reduction have no financial relationship with the tobacco industry, it is clear that the industry provides financial support to scientists who favor their position on harm reduction, some of whom have been particularly vocal ST advocates [38, 43]. Findings from tobacco industry documents clearly demonstrate that the motivation for industry funding of extramural research is to serve their business interests [85–87]. For example, not only has it been shown that industry-funded scientists were less likely to conclude that secondhand smoke had adverse health effects [88], but the industry actively funded individuals and institutions for the purpose of creating divisions within the public health community [89].

A bill has been passed by the U.S. House of Representatives [H.R. 1108] and another is currently pending in the U.S. Senate [S. 625] that would grant the U.S Food and Drug Administration (FDA) the authority to regulate the manufacturing, marketing and sale of tobacco products. Among its provisions, the legislation would require FDA approval before the introduction of tobacco products that are claimed to be “reduced harm.” Tobacco manufacturers would be required to provide scientific evidence that such products would reduce harm for individuals and for the population as a whole. The FDA also would have the authority to require changes in current and future tobacco products to protect public health. In the absence of a regulatory framework, which is the current status in the United States, it is largely left to the tobacco industry to decide what products will be introduced, their contents and toxin levels, their marketing, and the implicit claims that can be made. Perhaps when such decisions are under the regulatory control of public health authorities and not tobacco manufacturers it may be appropriate to discuss promotion of reduced harm tobacco products.

It is clear that more research on ST is needed, particularly regarding health risks among smokers who switch to exclusively using ST and the effectiveness of ST for long-term nicotine maintenance. Nevertheless, based on the precautionary principle widely used in public health [90], the burden of proof is on ST proponents to provide strong scientifically credible evidence to support their position. As we have outlined, such evidence currently is lacking, and it is therefore inappropriate at this time for the public health community to promote ST use as an evidence-based harm reduction strategy. Those considering ST use for harm reduction need to consider the entire body of research, along with the many practical and ethical challenges. We conclude that the implications extend far beyond the simple statement that “ST use is safer than cigarette smoking.”

## References

[1] Gartner CE, Hall WD, Vos T, Bertram MY, Wallace AK, Lim SS (2007). Assessment of Swedish snus for tobacco harm reduction: an epidemiological modeling study. Lancet.

[2] Stratton K, Shetty P, Wallace R, Bondurant S (2001). Clearing the Smoke: Assessing the science base for tobacco harm reduction.

[3] Royal College of Physicians of London Harm reduction in nicotine addiction: helping people who can’t quit.A report by the Tobacco Advisory Group of the Royal College of Physicians. Royal College of Physicians of London: London, UK, 2007. Available at: http://www.rcplondon.ac.uk/pubs/contents/4fc74817-64c5-4105-951e-38239b09c5db.pdf

[4] Rodu B, Godshall WT (2006). Tobacco harm reduction: an alternative cessation strategy for inveterate smokers. Harm Reduct. J.

[5] Kozlowski LT, O’Connor RJ, Edwards BQ (2003). Some practical points on harm reduction: what to tell your lawmaker and what to tell your brother about Swedish snus. Tob. Control.

[6] Whelan EM (2003). Cigarettes: what the warning label doesn’t tell you. The first comprehensive guide to the health consequences of smoking updated and revised for the 21st century.

[7] Brinson B (2006). Relative benefits: smokeless tobacco is thought to present less of a health risk than cigarettes; but many health advocates remain unconvinced. Tob. Reporter.

[8] Bates C, Fagerstrom K, Jarvis MJ, Kunze M, McNeill A, Ramstrom L (2003). European Union policy on smokeless tobacco: a statement in favour of evidence based regulation for public health. Tob. Control.

[9] Nelson DE, Mowery P, Tomar S, Marcus S, Giovino G, Zhao L (2006). Trends in smokeless tobacco use among adults and adolescents in the United States. Am. J. Public Health.

[10] Federal Trade Commission Smokeless tobacco report for the years 2002–2005 Federal Trade Commission Washington, D.C., U.S. 2007Available at http://www.ftc.gov/reports/tobacco/02-05smokeless0623105.pdf

[11] Statistics Sweden (2007). Use of alcohol and tobacco. Official Statistics of Sweden. Living Conditions Report No. 114..

[12] Braverman MT, Svendsen T, Lund KE, Aaro LE (2001). Tobacco use by early adolescents in Norway. Eur. J. Public Health.

[13] Substance Abuse and Mental Health Services Administration Results from the 2006 National Survey on Drug Use and Health: National Findings U.S. Department of Health and Human Services, Substance Abuse and Mental Health Services Administration, Office of Applied Studies Rockville, MD, U.S. 2007DHHS Publication No. SMA 07-4293.

[14] Hatsukami DK, Lemmonds C, Tomar SL (2003). Smokeless tobacco use: harm reduction or induction approach?. Prev. Med.

[15] Hatsukami DK, Henningfield JE, Kotlyar M (2004). Harm reduction approaches to reducing tobacco-related mortality. Annu. Rev. Public Health.

[16] Critchley JA, Unal B (2003). Health effects associated with smokeless tobacco: a systematic review. Thorax.

[17] Savitz DA, Meyer RE, Tanzer JM, Mirvish SS, Lewin F (2006). Public health implications of smokeless tobacco use as a harm reduction strategy. Am. J. Public Health.

[18] Tomar S (2005). Is smokeless tobacco effective in reducing cigarette smoking and tobacco-related harms? Member commentary on the smokeless tobacco debate. Soc. Res. Nicotine Tob. Newsletter.

[19] Luo J, Ye W, Zendehdel K, Adami J, Adami HO, Boffetta P, Nyrén O (2007). Oral use of Swedish moist snuff (snus) and risk for cancer of the mouth, lung, and pancreas in male construction works: a retrospective cohort study. Lancet.

[20] Boffetta P, Aagnes B, Weiderpass E, Andersen A (2005). Smokeless tobacco use and risk of cancer of the pancreas and other organs. Int. J. Cancer.

[21] Alguacil J, Silverman DT (2004). Smokeless and other noncigarette tobacco use and pancreatic cancer: a case-control study based on direct interviews. Cancer Epidemiol. Biomarker. Prev.

[22] International Agency for Research on Cancer (1985). Tobacco habits other than smoking. IARC Monog. Eval. Carcinog. Risk Chem. Hum. Vol. 37..

[23] International Agency for Research on Cancer (2007). Smokeless tobacco and some tobacco-related N-nitrosamines. IARC Monog. Eval. Carcinog. Risk Chem. Hum. Vol. 89.

[24] U.S. Department of Health and Human Services The health consequences of using smokeless tobacco. A report of the Advisory Committee to the Surgeon General. U.S. Department of Health and Human Services, Public Health Service Bethesda, MD, U.S. 1986NIH Publication No. 86-2874.

[25] National Toxicology Program (2005). Report on carcinogens.

[26] Levy DT, Mumford EA, Cummings KM, Gilpin EA, Giovino G, Hyland A, Sweanor D, Warner KE (2004). The relative risks of a low-nitrosamine smokeless tobacco product compared with smoking cigarettes: estimates of a panel of experts. Cancer Epidemiol. Biomarker. Prev.

[27] Richter P, Hodge K, Stanfill S, Zhang L, Watson C (2008). Surveillance of moist snuff: total nicotine, moisture, pH, un-ionized nicotine, and tobacco-specific nitrosamines. Nicotine Tob. Res.

[28] Stepanov I, Jensen J, Hatsukami D, Hecht SS (2008). New and traditional smokeless tobacco: Comparison of toxicant and carcinogen levels. Nicotine Tob. Res.

[29] Royal College of Physicians of London Protecting smokers, saving lives: the case for a tobacco and nicotine regulatory authority Royal College of Physicians of London London, U.K. 2002Available at http://www.rcplondon.ac.uk/pubs/books/protsmokers/index.asp

[30] Reynolds American Inc. Reynolds American CEO: ‘Progress Continues, Profits Climb’ [press release]. 27 April 2006. Available at http://www.reynoldsamerican.com/Newsroom/..%5Ccommon%5CViewPDF.aspx?postID=1128 Accessed on 13 March 2007.

[31] Altria Group, Inc.Remarks by Michael E. Szymanczyk, Chairman and CEO, Philip Morris USA Inc. at Prudential Consumer Conference. Boston, U.S., September 7, 2006. http://www.altria.com/media/press_release/03_02_pr_2006_09_07_01.asp Accessed on March 13, 2007.

[32] Philip Morris USA. Marlboro Snus fact sheet Available at http://www.philipmorrisusa.com/en/popup_marlboro_snus_fact_sheet.asp?source=home? Accessed on June 25, 2007.

[33] U.S. Smokeless Tobacco Company Magazine advertisement for Skoal® moist snuff: “Enjoy tobacco on a 4-hour flight? Absolutely.” 2005. Available at http://www.trinketsandtrash.org/tearsheet.asp?ItemNum=210630 Accessed on March 13, 2007.

[34] U.S. Smokeless Tobacco CompanyMagazine advertisement for Skoal® moist snuff:”Enjoy tobacco in a smoke-free sports bar? Believe it.” 2005. Available at http://www.trinketsandtrash.org/tearsheet.asp?ItemNum=210555 Accessed on March 13, 2007.

[35] Luik JC (2006). The truth that dare not speak: the moral case for smokeless tobacco. Tob. Report..

[36] Bolinder G, Alfredssom L, Englund A, deFaire U (1994). Smokeless tobacco use and increased cardiovascular mortality among Swedish construction workers. Am. J. Public Health.

[37] Henley SJ, Connell CJ, Richter P, Husten C, Pechacek T, Calle EE, Thun MJ (2007). Tobacco-related disease mortality among men who switched from cigarettes to spit tobacco. Tob. Control.

[38] Rodu B, Stier J (2005). Smokeout: not as easy as ABC. The Washington Times.

[39] LaVecchia C Lucchini F Negri E Boyle P Maisoneuve P Levi F Trends of cancer mortality in Europe, 1985–1989: II and IV Eur. J. Cancer 1992 28 514 599 *28A*, 1210–1281. 1627395 10.1016/0959-8049(92)90485-k

[40] Peto R, Lopez AD, Boreham J, Thun MJ, Health C (2006). Mortality from tobacco in developed countries: indirect estimation from national vital statistics. Lancet.

[41] U.S. Department of Health and Human Services (2004). The health consequences of smoking: a report of the Surgeon General.

[42] Kozlowski LT (2002). Harm reduction, public health, and human rights: smokers have a right to be informed of significant harm reduction options. Nicotine Tob. Res.

[43] Phillips CV, Guenzel B, Bergen P (2005). Deconstructing anti-harm reduction metaphors: mortality risk from falls and other traumatic injuries compared to smokeless tobacco use. Harm Reduct. J.

[44] Weinstein ND, Marcus SE, Moser RP (2005). Smokers’ unrealistic optimism about their risk. Tob. Control.

[45] Petty RE, Cacioppo JT (1986). Communication and persuasion: central and peripheral routes to attitude change.

[46] Slovic P (2001). Smoking: Risk, Perception and Policy..

[47] Tomar SL (2002). Association between smoking and snuff use in U.S. men: implications for harm reduction. Am. J. Prev. Med.

[48] Bombard JM, Pederson LL, Nelson DE, Malarcher AM (2007). Are smokers only using cigarettes: exploring current polytobacco use among an adult population. Addict. Behav.

[49] Foulds J, Ramstrom L, Burke M, Fagerstrom K (2003). Effect of smokeless tobacco (snus) on smoking and public health in Sweden. Tob. Control.

[50] Furberg H, Bulik CM, Lerman C, Lichtenstein P, Pederson NL, Sullivan PF (2005). Is Swedish snus associated with smoking initiation or smoking cessation. Tob. Control.

[51] Ramstrom LM, Foulds J (2006). Role of snus in initiation and cessation of tobacco smoking in Sweden. Tob. Control.

[52] Tomar SL (2007). Epidemiological perspectives on smokeless tobacco marketing and population harm. Am. J. Prev. Med.

[53] Novotny TE, Pierce JP, Fiore MC, Davis RM (1989). Smokeless tobacco use in the United States: the adult use of tobacco surveys. NCI Monogr.

[54] Kozlowski LT, O’Connor RJ, Edwards BQ, Flaherty BP (2003). Most smokeless tobacco use is not a causal gateway to cigarettes: using order of product use to evaluate causation in a national U.S. sample. Addiction.

[55] Tomar SL, Loree M (2004). Errors in analyzing associations between use of smokeless tobacco and cigarettes. Addiction.

[56] Tilashalski K, Rodu B, Cole P (1998). A pilot study of smokeless tobacco in smoking cessation. Am. J. Med.

[57] Tilashalski K, Rodu B, Cole P (2005). Seven year follow-up of smoking cessation with smokeless tobacco. J. Psychoactive Drugs.

[58] Tønnesen P, Mikkelsen K, Bremann L (2008). Smoking cessation with smokeless tobacco and group therapy: an open, randomized, controlled trial. Nicotine Tob. Res.

[59] Fiore MC, Bailey WC, Cohen SJ (2000). Treating tobacco use and dependence.

[60] Office of National Drug Control PolicyMethadone [fact sheet]. White House, Office of National Drug Control Policy, Drug Policy Information Clearinghouse: Rockville, MD., U.S., 2000. Available at www.whitehousedrugpolicy.gov/publications/factsht/methadone/index.html Accessed on 20 November 2007.

[61] Djordjevic MV, Hoffmann D, Glynn T, Connolly GN (1995). US commercial brands of moist snuff, 1994. I. Assessment of nicotine, moisture, and pH. Tob. Control.

[62] Henningfield J, Radzius A, Cone E (1995). Estimation of available nicotine content in six smokeless tobacco products. Tob. Control.

[63] Henningfield JE, Rose CA, Giovino GA (2002). Brave new world of tobacco disease prevention: promoting dual tobacco-product use?. Am. J. Prev. Med.

[64] Johnston LD O’Malley PM Bachman JG Schulenber JE Monitoring the Future: national results on adolescent drug use. Overview of key findings, 2006 U.S. Department of Health and Human Services, National Institutes of Health, National Institute on Drug Abuse Bethesda, MD, U.S. 2007 NIH Publication No. 07-6202.

[65] Ary DV, Lichtenstein E, Severson HH (1987). Smokeless tobacco use among male adolescents: patterns, correlates predictors and the use of other drugs. Prev. Med.

[66] Ary DV (1989). Use of smokeless tobacco among male adolescents: concurrent and prospective relationships. NCI Monogr.

[67] Dent CW, Sussman S, Johnson CA, Hansen WB, Flay BR (1987). Adolescent smokeless tobacco incidence: relationship with other drugs and psychosocial variables. Prev. Med.

[68] Glover ED, Laflin M, Edwards SW (1989). Age of initiation and switching patterns between smokeless tobacco and cigarettes among college students in the United States. Am. J. Public Health.

[69] Tomar SL (2003). Is use of smokeless tobacco a risk factor for cigarette smoking? The U.S. experience. Nicotine Tob. Res.

[70] O’Connor RJ, Flaherty BP, Quinio Edwards B, Kozlowski LT (2003). Regular smokeless tobacco use is not a reliable predictor of smoking onset when psychosocial predictors are included in the model. Nicotine Tob. Res.

[71] Tomar SL (2003). Smokeless tobacco use is a significant predictor of smoking when appropriately modeled. Nicotine Tob. Res.

[72] Severson HH, Forrester KK, Biglan A (2007). Use of smokeless tobacco is a risk factor for cigarette smoking. Nicotine Tob Res.

[73] Haddock CK, Vander Weg M, DeBon M, Klesges RC, Talcott GW, Lando H, Peterson A (2001). Evidence that smokeless tobacco use is a gateway for smoking initiation in young adult males. Prev. Med.

[74] Myers M Statement of Matthew Myers, President, Campaign for Tobacco-Free Kids, on Smokeless (Spit) Tobacco Before the U.S. House Energy and Commerce CommitteeSubcommittee on Commerce, Trade and Consumer Protection, June 3, 2003. Available at http://tobaccofreekids.org/reports/spit/MyersTestimony.pdf Accessed on April 24, 2007.

[75] Campaign for Tobacco Free Kids (2006). A Broken Promise to our Children. The 1998 State Tobacco Settlement Eight Years Later.

[76] Gartner CE, Hall WD, Chapman S, Freeman B (2007). Should the health community promote smokeless tobacco (snus) as a harm reduction measure?. PLoS Med.

[77] Antonsson AB (1995). Substitution of dangerous chemicals—the solution to problems with chemical hazards in the work environment?. Am. Ind. Hyg. Assoc. J.

[78] Fischer K Schot J, Eds. Environmental strategies for industry: international perspectives on research needs and implications. Island Press Washington, D.C., U.S. 1993

[79] Rothman AJ, Kiviniemi MT (1999). Treating people with information: an analysis and review of approaches to communicating health risk information. J. Natl. Cancer Inst. Monogr.

[80] National Cancer Institute Risks associated with smoking cigarettes with low machine-measured yields of tar and nicotine.Smoking and Tobacco Control Monographs No. 13. U.S. Department of Health and Human Services, National Institutes of Health, National Cancer Institute Bethesda, MD, U.S. 2001 NIH Pub. No 02-5074.

[81] Kahneman D, Tversky A, Slovic P (1982). Judgment under uncertainty: heuristics and biases..

[82] Remington PL, Nelson DE, Parvanta C (2002). Communicating public health information effectively: a guide for practitioners..

[83] United States District Court for The District of Columbia. United States of America *et al*. v. Philip Morris USA, Inc.Civil Action No. 99-2496 (GK). Final Opinion. United States District Court for The District of Columbia Washington, DC, U.S. 1999 Available at: http://tobaccofreekids.org/reports/doj/FinalOpinion.pdf Accessed on June 25, 2007.

[84] Ling PM, Glantz SA (2005). Tobacco industry consumer research on socially acceptable cigarettes. Tob. Control.

[85] Barnoya J, Glantz SA (2006). The tobacco industry’s worldwide ETS consultants project: European and Asian components. Eur. J. Public Health.

[86] Bero LA (2005). Tobacco industry manipulation of research. Public Health Rep.

[87] Parascandola M (2005). Science, industry, and tobacco harm reduction: a case study of tobacco industry scientists’ involvement in the National Cancer Institute’s Smoking and Health Program, 1964–1980. Public Health Rep.

[88] Muggli ME, Forster JL, Hurt RD, Repace JL (2001). The smoke you don’t see: uncovering tobacco industry scientific strategies aimed against environmental tobacco smoke policies. Am. J. Public Health.

[89] McDaniel PA, Smith EA, Malone RE (2006). Philip Morris’s Project Sunrise: weakening tobacco control by working with it. Tob. Control.

[90] Cranor CF (2004). Toward understanding aspects of the precautionary principle. J. Med. Philos.

